# Rapidly Progressive Buccal Hematoma Following Local Anesthetic Injection: A Case Report

**DOI:** 10.3390/reports8020088

**Published:** 2025-06-05

**Authors:** Solon Politis, Dimitris Tatsis, Asterios Antoniou, Alexandros Louizakis, Konstantinos Paraskevopoulos

**Affiliations:** Department of Oral & Maxillofacial Surgery, Aristotle University of Thessaloniki, Specialized Cancer Treatment and Reconstruction Centre, General Hospital of Thessaloniki “George Papanikolaou”, 57010 Thessaloniki, Greece; solonpolitis88@gmail.com (S.P.); ast.antoniou@gmail.com (A.A.); alexluimd@gmail.com (A.L.); kostparas@yahoo.gr (K.P.)

**Keywords:** airway compromise, buccal hematoma, case report, dental procedure, facial artery, local anesthetic, multidisciplinary management, surgical decompression

## Abstract

**Background and Clinical Significance:** Local anesthetic injections, routine in dental practice, ensure pain control during procedures like root canal treatments. Though generally safe, they can occasionally cause hematomas, localized blood accumulations in tissue planes. Rapidly expanding hematomas in the head and neck are exceptionally rare but dangerous due to anatomical complexity, potentially threatening the airway. This case report emphasizes the critical need for the prompt recognition and management of such complications to prevent life-threatening outcomes, highlighting vigilance in routine dental procedures. **Case Presentation**: A 63-year-old male presented with rapidly enlarging right buccal swelling four hours post-local anesthetic injection for a root canal on a right maxillary molar. Examination showed warm, erythematous edema and buccal ecchymosis; a CT scan confirmed a 3.8 cm × 8.4 cm × 5.5 cm buccal space hematoma. His medical history revealed controlled type 2 diabetes and hyperlipidemia, and his coagulation was normal. Conservative management failed as the hematoma progressed, limiting mouth and eye opening. Urgent surgical decompression under general anesthesia evacuated clots and ligated facial and angular arteries. ICU monitoring ensured airway stability, with discharge on day three with antibiotics and follow-up. **Conclusions**: This case highlights the rare potential for dental anesthetic injections to cause rapidly progressive hematomas, requiring urgent surgical intervention and multidisciplinary care to prevent airway compromise. Early recognition, imaging, and decisive management are vital in achieving favorable outcomes in such serious complications.

## 1. Introduction and Clinical Significance

The administration of local anesthetic injections in the oral cavity represents a cornerstone of modern dental practice, offering a safe and effective means of achieving targeted anesthesia to facilitate a wide array of procedures [1]. This operative technique, which delivers anesthetic agents directly into specific sites within the oral mucosa or surrounding tissues, is lauded for its precision and reliability in mitigating pain during interventions such as root canal treatments, tooth extractions, and minor surgical procedures [1]. Despite its well-established safety profile, the procedure is not devoid of risks, as any invasive manipulation of human tissues carries the potential for complications. Among these, hematoma formation stands out as a recognized, albeit rare, adverse event that can arise following the infiltration of anesthetic solutions [2,3]. Hematomas, characterized by localized collections of extravasated blood within tissue planes, may occur independently of or in association with underlying coagulation or platelet dysfunction, complicating their clinical presentation and management [4,5]. The rarity of this complication is well documented in the literature, with a paucity of reported cases highlighting its occurrence in the context of routine dental procedures [6,7].

While hematomas resulting from local anesthetic injections are typically benign and self-limiting, their potential to escalate into critical clinical scenarios cannot be understated. In exceptional cases, such hematomas may exhibit rapid and aggressive expansion, infiltrating deeper anatomical spaces within the head and neck, including the pterygomandibular, parapharyngeal, or retropharyngeal spaces [8,9]. Such progression poses a significant threat to patient safety, as the encroachment of a hematoma into these critical regions can compromise vital structures, most notably the airway, leading to life-threatening consequences such as asphyxiation [10,11]. The likelihood of such an outcome is heightened by the unique anatomical complexity of the head and neck, where compact tissue planes and proximity to critical neurovascular structures amplify the potential for rapid clinical deterioration. Consequently, cases requiring hospitalization, particularly in an intensive care unit (ICU) setting, are exceedingly uncommon and represent a critical departure from the typical clinical course of post-anesthetic hematomas. This rarity underscores the importance of documenting and analyzing such cases to enhance clinical awareness and inform evidence-based management strategies.

Clinical Significance: This is a rare but potentially life-threatening complication of a rapidly expanding buccal hematoma following a routine dental anesthetic injection, emphasizing the need for prompt recognition, urgent surgical intervention, and multidisciplinary management to prevent severe outcomes such as airway compromise.

## 2. Case Presentation

A 63-year-old male patient was urgently referred to the Emergency Department of our tertiary care medical facility, presenting with an acute, progressively enlarging right buccal swelling. This distressing symptom manifested approximately four hours subsequent to the administration of a local anesthetic injection, which was employed to facilitate a root canal procedure on a right maxillary molar. Upon arrival, a meticulous physical examination was conducted, revealing a warm, erythematous edema localized to the right infraorbital region, extending distally to the peripheral boundaries of the buccal area. Additionally, the right buccal mucosa exhibited pronounced ecchymosis, suggestive of underlying hemorrhagic activity. Clinical differential diagnosis included hematoma, traumatic injury, allergic edema, infection, or a vascular anomaly, with the latter two having a far lower possibility of being the final diagnosis of the patient.

**Figure 1:**
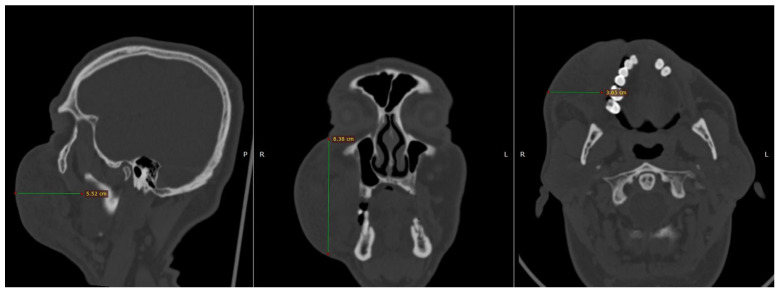
Computed tomography (CT), which was performed in the Emergency Department. The maximum dimensions of the lesion in the right buccal area measured 3.8 × 8.4 × 5.5 cm.

To elucidate the nature and extent of the observed pathology, the patient underwent an urgent computed tomography (CT) scan of the craniofacial region. The imaging study disclosed a substantial hyperdense lesion within the right buccal space, with dimensions measuring 3.8 cm × 8.4 cm × 5.5 cm, as depicted in Figure 1. This radiographic finding corroborated the clinical suspicion of a significant hematoma.

A comprehensive review of the patient’s medical history revealed a diagnosis of type 2 diabetes mellitus, managed pharmacologically, alongside hyperlipidemia, similarly controlled with medication. Notably, the patient denied any history of drug allergies, tobacco use, or alcohol consumption, factors that could otherwise predispose him to complications in such clinical scenarios. Laboratory investigations, including a complete coagulation profile, demonstrated normal parameters, with a platelet count well within the reference range, thereby excluding coagulopathy as a contributing factor to the hematoma formation (platelets, 170,000/μL; prothrombin time, PT 12 s; and activated partial thromboplastin time, APTT 27 s).

Based on clinical and radiographic findings, a diagnosis of right buccal hematoma was established. Initially, in the first hours after the admission, a conservative management strategy was adopted, prioritizing close observation and the initiation of intravenous chemoprophylaxis to mitigate the risk of secondary infection within the hematoma. However, during the subsequent clinical course, and specifically 4 h after the admission, the hematoma exhibited alarming progression, with further expansion involving the lower eyelid, manifesting as edema and ecchymosis. Concomitantly, the patient developed restricted mouth opening and diminished strength in right eye opening, indicative of an actively expanding hematoma exerting pressure on adjacent anatomical structures.

**Figure 2:**
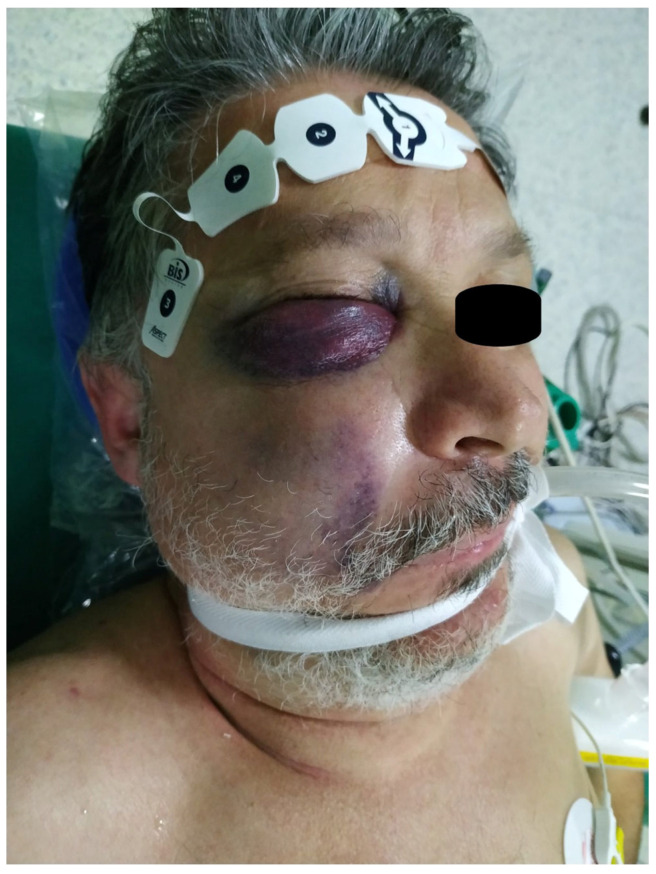
The patient was intubated in the operation room. A right buccal and a right lower eyelid swelling with ecchymosis were present. The swelling seemed to be an active and expanding hematoma under tension.

The progressive nature of the hematoma raised significant concerns regarding potential encroachment into critical anatomical spaces. Specifically, there was a plausible risk that the intratissue hemorrhage could transgress the posterior boundary of the buccal space, infiltrate the pterygomandibular space, and ultimately extend into the parapharyngeal space. Such an eventuality posed a grave threat to the patient’s airway integrity, with the potential for catastrophic asphyxiation if left unaddressed. Consequently, an urgent decision was made to proceed with surgical intervention under general anesthesia, as illustrated in Figure 2.

During the surgical decompression procedure, multiple blood clots were meticulously evacuated from the affected region. Intraoperative exploration identified two arterial branches, namely, the facial and angular arteries, as the primary sources of the hemorrhage. The vascular walls of these arteries appeared compromised, necessitating their ligation to achieve hemostasis. The procedure was executed with precision to minimize further tissue trauma and ensure optimal outcomes.

Following the surgical intervention, the patient was transferred to the intensive care unit (ICU) to facilitate rigorous postoperative monitoring, with particular emphasis on maintaining hemodynamic stability and safeguarding airway patency. On the second postoperative day, the patient was successfully decannulated, marking a significant milestone in his recovery. He was subsequently transferred to a general ward, where his clinical course remained uneventful. A thorough postoperative evaluation of coagulation parameters was conducted, reaffirming the absence of any underlying coagulopathy.

**Figure 3:**
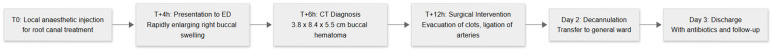
Timeline of the patient’s clinical course. T0 was the time of the local anesthetic injection, and the progression of hematoma is described initially hourly and daily until the discharge of the patient from the hospital.

By the third postoperative day, the patient’s condition had stabilized sufficiently to warrant discharge from the hospital. He was prescribed oral antimicrobial therapy to prevent postoperative infections and was provided detailed instructions for routine follow-up evaluations to monitor his recovery and ensure the absence of recurrent complications. The patient’s adherence to the prescribed regimen and follow-up schedule was emphasized to optimize long-term outcomes. The patient’s clinical course timeline is summarized in Figure 3.

## 3. Discussion

Hematomas most commonly result from inadvertent trauma to vascular structures during needle insertion, particularly branches of the facial or maxillary arteries [3,12,13]. The extent of hematoma formation is influenced by the injured vessel’s caliber, the surrounding tissue density, and the patient’s coagulation profile [2,4]. In the current case, trauma to the facial and angular arteries resulted in a rapidly expanding hematoma, despite the absence of coagulopathy or platelet dysfunction [5,14]. This confirms that direct vascular insult is often the primary etiological factor in such cases. Nevertheless, there have been cases with rapidly expanding hematomas related to endodontic treatments [15,16,17].

The buccal space, a frequent site for such hematomas, is bordered by fascial planes that allow extension into adjacent compartments such as the pterygomandibular, parapharyngeal, and even submandibular spaces [8,18]. These anatomical continuities increase the risk of airway compromise if hematomas expand unchecked [19]. Cases involving the parapharyngeal space are particularly concerning, as pressure on the pharynx or larynx may lead to airway obstruction [9,20,21]. In this patient, hematoma extension into the infraorbital area and signs of trismus and periorbital swelling prompted urgent surgical intervention to pre-empt airway involvement.

Patients with post-anesthetic hematomas may present with localized pain, swelling, ecchymosis, and varying degrees of functional impairment such as trismus or ocular involvement [7,22]. In this case, the patient developed severe facial swelling and restricted mouth opening, accompanied by compromised eye opening—signs indicative of deeper anatomical spread and mechanical compression. These findings necessitated imaging with CT, which revealed a hyperdense area in the buccal area, which was consistent with a substantial hematoma (3.8 cm × 8.4 cm × 5.5 cm), ruling out other causes such as infection or allergic reactions [6,23,24].

A differential diagnosis in such presentations must consider allergic edema, infections, or vascular anomalies; however, the rapid progression and absence of systemic signs often point toward hematoma [25,26]. In the present case, swelling, erythema, ecchymosis, and functional impairments (restricted mouth and eye opening) emerged shortly after the dental visit (four hours post-injection), suggesting a hematoma. The CT scan confirmed a hyperdense buccal space lesion. Surgical decompression revealed bleeding from the facial and angular arteries, confirming vascular trauma from the injection as the cause. The patient’s normal coagulation parameters excluded bleeding disorders. Differential diagnoses, including allergic edema, infection, vascular anomalies, and irrigant extrusion, were ruled out due to there being no systemic allergic symptoms, fever, or prior vascular history and the rapid onset being inconsistent with irrigant-related damage, which typically causes immediate pain or chemical irritation. These findings underscore the injection as the etiological factor, highlighting the need for prompt recognition and management.

Small, non-expanding hematomas are generally managed conservatively using cold compresses, pressure application, and observation [2]. However, progressive or symptomatic hematomas—especially those that threaten the airway—require prompt surgical decompression [7,16]. In the present case, conservative approaches failed, and surgical evacuation was performed under general anesthesia. Intraoperative findings confirmed active bleeding from branches of the facial and angular arteries, which were successfully ligated—an approach consistent with other reports [22,27].

Postoperative ICU monitoring, including prolonged intubation, was critical in managing the potential for delayed swelling or rebleeding, which can reintroduce airway risks [10,11]. This stepwise escalation in management reflects a patient-specific, risk-based approach supported by the literature.

Preventing hematomas requires precise injection techniques, including aspiration before deposition and avoiding excessive depth or angulation in the needle [2,18]. While hematomas are not entirely preventable, their incidence can be reduced through careful preoperative assessment, especially in patients with known bleeding tendencies or those on anticoagulants [5]. Awareness of at-risk anatomical regions and the early recognition of unusual post-procedural swelling can significantly influence outcomes.

This case report has several strengths. It documents a rare, life-threatening complication of a routine dental anesthetic injection, contributing to the limited literature on rapidly progressive buccal hematomas. The detailed clinical description, supported by CT imaging and intraoperative findings, provides a clear diagnostic and management pathway for clinicians. The case underscores the value of multidisciplinary collaboration, involving oral and maxillofacial surgeons, anesthetists, and critical care specialists, to ensure prompt, comprehensive care for complex cases with potential airway involvement [8]. However, our report has limitations. As a single case, its findings may not be broadly generalizable. The exact mechanism behind rapid hematoma progression remains unclear, as the report focuses on a single patient without comparative analysis to other cases in the literature. Finally, while preventive measures are discussed, their efficacy cannot be evaluated in this case, limiting specific guidance for clinical practice in dentistry.

## 4. Conclusions

Though infrequent, hematoma formation following local anesthesia can evolve into a life-threatening condition due to the anatomical complexity of the head and neck. The present case illustrates how rapid hematoma progression, involving high-risk anatomical spaces, necessitates urgent surgical and critical care intervention. Early recognition, appropriate imaging, and surgical decompression—combined with vigilant postoperative airway management—are key in preventing adverse outcomes. This case serves as a stark reminder that even routine dental procedures require clinical vigilance and readiness to manage rare but serious complications.

## Data Availability

The original contributions presented in this study are included in the article. Further inquiries can be directed to the corresponding author.

## Abbreviations

The following abbreviations are used in this manuscript:

ICU	Intensive care unit
